# Experimental infection of Cynomolgus Macaques with highly pathogenic H5N1 influenza virus through the aerosol route

**DOI:** 10.1038/s41598-018-23022-0

**Published:** 2018-03-19

**Authors:** Tokiko Watanabe, Kiyoko Iwatsuki-Horimoto, Maki Kiso, Noriko Nakajima, Kenta Takahashi, Tiago Jose da Silva Lopes, Mutsumi Ito, Satoshi Fukuyama, Hideki Hasegawa, Yoshihiro Kawaoka

**Affiliations:** 1 Division of Virology, Department of Microbiology and Immunology, Institute of Medical Science, University of Tokyo, Tokyo, 108-8639 Japan; 2 Department of Pathology, National Institute of Infectious Diseases, Shinjuku-ku, Tokyo, 162-8640 Japan; 3 Influenza Research Institute, Department of Pathobiological Sciences, School of Veterinary Sciences, University of Wisconsin-Madison, Madison, WI 53711 USA; 4 Department of Special Pathogens, International Research Center for Infectious Diseases, Institute of Medical Science, University of Tokyo, Minato-ku, Tokyo, 108-8639 Japan

## Abstract

Several animal models are used to study influenza viruses. Intranasal inoculation of animals with a liquid inoculum is one of the main methods used to experimentally infect animals with influenza virus; however, this method does not reflect the natural infection with influenza virus by contact or aerosol route. Aerosol inhalation methods have been established with several influenza viruses for mouse and ferret models, but few studies have evaluated inoculation routes in a nonhuman primates (NHP) model. Here, we performed the experimental infection of NHPs with a highly pathogenic H5N1 influenza virus via the aerosol route and demonstrated that aerosol infection had no effect on clinical outcome, but caused broader infection throughout all of the lobes of the lung compared with a non-aerosolized approach. Aerosol infection therefore represents an option for inoculation of NHPs in future studies.

## Introduction

Influenza viruses cause annual epidemics and periodic pandemics. We have experienced three pandemics in the 20^th^ century (i.e., Spanish flu in 1918–1919, Asian influenza in 1957, and Hong Kong influenza in 1968) and one in the 21^st^ century (i.e., pandemic influenza A (H1N1) 2009 in 2009). These influenza pandemics have a huge impact on public health and the global economy^1^. Additionally, recent sporadic human infections with H5N1 and H7N9 avian influenza viruses have raised the pandemic threat of these viruses^2–5^. The continued circulation of H5N1 viruses in birds provides opportunities for them to infect humans. Indeed, human cases of H5N1 infections have been reported in several countries, with a total of 860 confirmed cases and 454 fatalities as of 30 Oct 2017, which is a case fatality rate of approximately 53% (http://www.who.int/influenza/human_animal_interface/2017_10_30_tableH5N1.pdf?ua=1). Therefore, concern over the pandemic potential of H5N1 viruses is clearly justified.

Influenza virus can be transmitted from person to person through several transmission modes, including direct contact, indirect contact with a contaminated object (fomite), droplet transmission (droplets of >5 µm in diameter), and droplet nuclei transmission, also referred as airborne or aerosol transmission (droplet nuclei of <5 µm in diameter, which can remain suspended in the air for prolonged periods). Experimental infection of animal models with influenza viruses is an essential component of the characterization of influenza viruses. Mice, ferrets, and nonhuman primates (NHPs) are commonly used to examine the pathogenicity, replicative ability, and transmissibility of influenza viruses, although each model has its advantages and limitations. NHPs have been frequently used as a model to characterize the virulence of various influenza viruses, such as highly pathogenic H5N1, and the 1918 and 2009 pandemic viruses, because of their close genetic relationship to humans^6–8^. Intranasal and/or intratracheal inoculation with a liquid inoculum of influenza viruses has been the primary method used to infect NHPs; however, a liquid inoculum does not reflect the natural infection.

Aerosol inhalation methods for inoculation of mice and ferrets have been established with several influenza viruses, including H5N1 viruses^9–13^. In the ferret model, these studies demonstrated that the inoculation of animals with highly pathogenic avian influenza H5N1 virus via the aerosol route led to higher nasal wash virus titers, earlier onset of clinical signs, and/or a broader spectrum of disease compared with infection via intranasal inoculation despite no difference in lethality^9–11^. In a murine model, Belser *et al*.^12^ demonstrated that inoculation of mice with H5N1, H7N9, or influenza A(H1N1)pdm2009 viruses via the aerosol or intranasal route resulted in comparable levels of morbidity and mortality among the infected mice. In contrast, few reports have evaluated inoculation routes in the NHP model. A recent study demonstrated that challenge of NHPs with pandemic 2009 viruses (A/California/04/2009; H1N1pdm) through the aerosol route resulted in efficient distribution of virus in both the upper and lower respiratory tracts, whereas intranasal infection led to limited distribution of virus in the upper respiratory tract^14^. Wonderlich *et al*.^15^ recently established aerosol infection with highly pathogenic H5N1 influenza virus in the NHP model; however, they have not compared the outcomes in the NHPs infected via the aerosol route with those in NHPs infected via the conventional method of liquid virus inoculation. Therefore, in this study, we compared the virus distribution, pathogenicity, and disease outcome in NHPs infected via the aerosol route with these parameters in NHPs infected through multiple routes (e.g., the intranasal and intratracheal routes).

## Results

### Establishment of an H5N1 virus infection system in nonhuman primates through an aerosol route by using a commercial nebulizer

A wide range of commercial nebulizers are available for clinical and research use. In this study, we used the ultrasonic nebulizer NE-U17 from Omron Healthcare Co, Ltd, Japan. This was chosen because it is easy to use and could reduce the treatment time needed compared with other nebulizer devices^16^, thereby making the procedure less stressful for the animals. The particle size is also critical for aerosol inhalation because aerosol transmission (also referred to as droplet nuclei transmission) occurs when small particles (<5 µm) are inhaled. A previous study demonstrated that the NE-U17 nebulizer generates a large fine particle fraction of aerosol mass <5 µm (the mean percentage of the fine particle fraction was 46%)^16^.

We used 2- to 3-year-old male cynomolgus macaques that weighed 2–3 kg each. Four animals were inoculated with 4 ml of a 10^7^ PFU/ml solution of the highly pathogenic H5N1 avian influenza virus A/Vietnam/UT3040/2004 strain (VN3040) through the aerosol route by using the ultrasonic nebulizer NE-U17. These animals were defined as ‘the aerosol method group’. As a control, 4 ml of the virus solution was administered through multiple routes by the conventional method, that is, by the intranasal (0.4 ml each to the left and right nostril), intraoral (0.4 ml each to the left and right tonsil), intraocular (0.1 ml per eye), and intratracheal (2.2 ml using a tracheal catheter) routes (defined as ‘the conventional method group’). The virus dose of 4 × 10^7^ PFU of influenza virus was chosen for inoculation because a similar virus dose has been widely used in previous studies to infect NHPs to ensure the greatest likelihood of virus infection and replication in the inoculated animals^6,15,17–19^.

**Figure 1:**
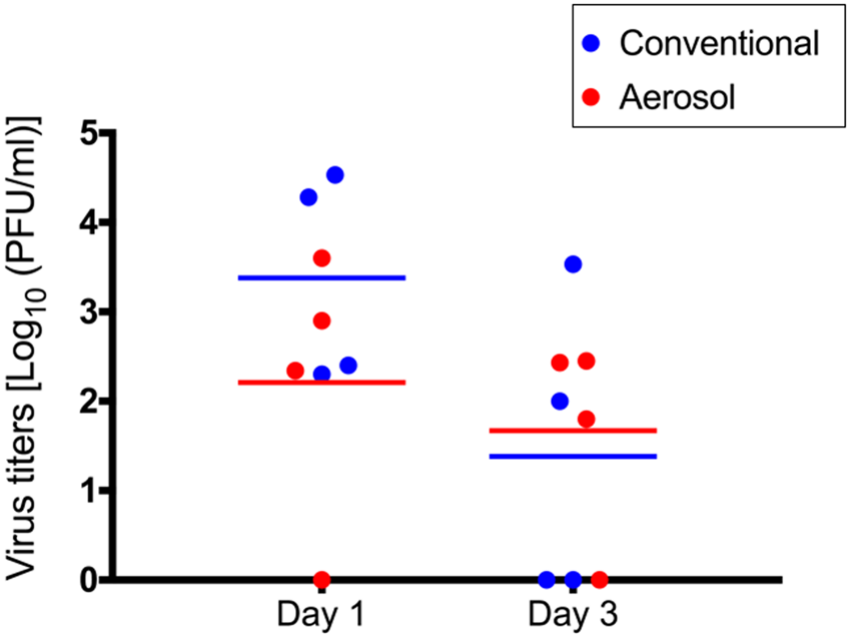
Virus titers in respiratory swabs from infected cynomolgus macaques. Cynomolgus macaques were inoculated with 4 ml of a 10^7^ PFU/ml solution of the highly pathogenic H5N1 avian influenza virus A/Vietnam/UT3040/2004 strain (VN3040) through the aerosol route by using the ultrasonic nebulizer NE-U17 (defined as “the aerosol method group”). As a control, 4 ml of the virus solution was administered through multiple routes, which is the conventional method; by intranasal (0.4 ml each to the left and right nostril), intraoral (0.4 ml each to the left and right tonsil), intraocular (0.1 ml per eye), and intratracheal (2.2 ml using a tracheal catheter) routes (defined as “the conventional method group”). Nasal swab samples were collected on days 1 and 3 post-infection for virus titration. Red and blue horizontal bars show the mean titers for the aerosol and conventional method groups, respectively.

Upon virus infection, none of the animals exhibited noticeable clinical signs (i.e., no appreciable body weight loss or fever was observed in any of the infected animals) except for a slight decrease in appetite among all of the infected animals. To examine virus shedding, nasal swab samples were collected from the infected animals on days 1 and 3 post-infection for virus titration. On day 1 post-infection, VN3040 virus was recovered from four animals in the conventional method group, whereas viruses were detected from three of four animals in the aerosol method group (Table 1 and Fig. 1). The mean virus titers were not significantly different between the conventional and the aerosol method groups [3.38 and 1.9 log_10_ (PFU/ml), respectively] (Fig. 1). On day 3 post-infection, VN3040 virus was recovered from nasal swabs of two and three animals in the conventional and aerosol method groups, respectively, and the mean virus titers were comparable between the two groups.

**Table 1:** Virus titers in nasal swabs from cynomolgus macaques infected with the H5N1 virus via the conventional or aerosol method^a^.

Animal ID	Virus titers (log_10_ PFU/ml) of animals infected with VN3040 virus via the:							
	Conventional method				Aerosol method			
	#1–4	#2–2	#3–1	#3–2	#1–9	#2–3	#3–3	#3–4
Day 1	2.40	2.30	4.53	4.28	3.60	—	2.90	2.34
Day 3	—	3.53	2.00	—	1.80	—	2.45	2.43

^a^Cynomolgus macaques were inoculated with 4 ml of a 10^7^ PFU/ml solution of A/Vietnam/UT3040/2004 virus (VN3040) by the conventional method (through a combination of the intratracheal, intranasal, ocular, and oral routes) or via the aerosol method by using a nebulizer.

^b^—, virus not detected (detection limit: 1.3 log_10_ PFU/ml).

### H5N1 virus replicates across a larger area of the lower respiratory tract of animals infected via the aerosol method compared to infection via the conventional method

To compare the distribution of H5N1 virus in animals infected via the conventional method with that in animals infected via the aerosol method, animals from each group were euthanized on day 4 post-infection and organ samples were collected for virus titration. Relatively high virus titers were detected in the tonsils of all infected animals in both groups (Table 2). Viruses were recovered from the nasal mucosa, trachea, and bronchus of some of the infected animals in both groups (Table 2). Additionally, virus was recovered from the mediastinal lymph nodes of two and one of four animals in the conventional and aerosol method groups, respectively. Also, virus was detected in the duodenum and brain of one of four animals in the conventional and aerosol method groups, respectively (Table 2).

**Table 2:** Virus titers in the organs of cynomolgus macaques infected with H5N1 virus via the conventional or aerosol method^a^

Animal ID	Virus titers (log_10_ PFU/g) of animals infected with VN3040 virus via the:							
	Conventional method				Aerosol method			
	#1–4	#2–2	#3–1	#3–2	#1–9	#2–3	#3–3	#3–4
Nasal mucosa	—^b^	—	—	3.27	4.58	—	3.00	—
Tonsils	6.82	5.43	6.00	5.77	5.58	5.20	6.87	4.22
Trachea	6.04	3.51	3.33	2.60	5.02	—	2.15	—
Bronchus (right)	4.03	—	3.10	3.08	4.55	3.36	3.74	4.26
Bronchus (left)	4.52	—	3.78	3.00	4.26	—	3.60	2.70
Lung (upper right)	—	—	3.99	3.78	3.96	3.70	4.58	5.00
Lung (middle right)	—	—	3.91	3.60	4.16	3.97	4.93	4.88
Lung (lower right)	—	4.77	4.77	4.51	3.90	4.05	6.71	4.92
Lung (upper left)	—	—	3.54	2.40	4.05	4.02	6.50	4.40
Lung (middle left)	—	2.30	4.08	—	4.35	4.06	4.40	3.94
Lung (lower left)	5.37	3.30	5.23	5.11	4.73	3.94	4.54	3.48
Brain (frontal)	NT^c^	—	—	—	NT	—	—	—
Brain (parietal)	NT	—	—	—	NT	—	—	—
Brain (temporal)	NT	—	—	—	NT	—	—	—
Brain (occipital)	NT	—	—	—	NT	—	2.15	—
Brain (cerebellum)	NT	—	—	—	NT	—	—	—
Brain (brain stem)	NT	—	—	—	NT	—	—	—
Olfactory bulb	NT	—	—	—	NT	—	—	—
Mediastinal								
lymph node	NT	—	3.85	2.19	NT	—	2.70	—
Heart	NT	—	—	—	NT	—	-	—
Spleen	NT	—	—	—	NT	—	—	—
Kidney	NT	—	—	—	NT	—	—	—
liver	NT	—	—	—	NT	—	—	—
Duodenum	NT	—	1.92	—	NT	—	—	—
Rectum	NT	—	—	—	NT	—	—	—

^a^Cynomolgus macaques were inoculated with 4 ml of a 10^7^ PFU/ml solution of VN3040 virus by the conventional method (through a combination of the intratracheal, intranasal, ocular, and oral routes) or via the aerosol method by using a nebulizer.

^b^—, virus not detected (detection limit: 1.3 log_10_ PFU/ml/organ piece).

^c^NT, not tested.

**Figure 2:**
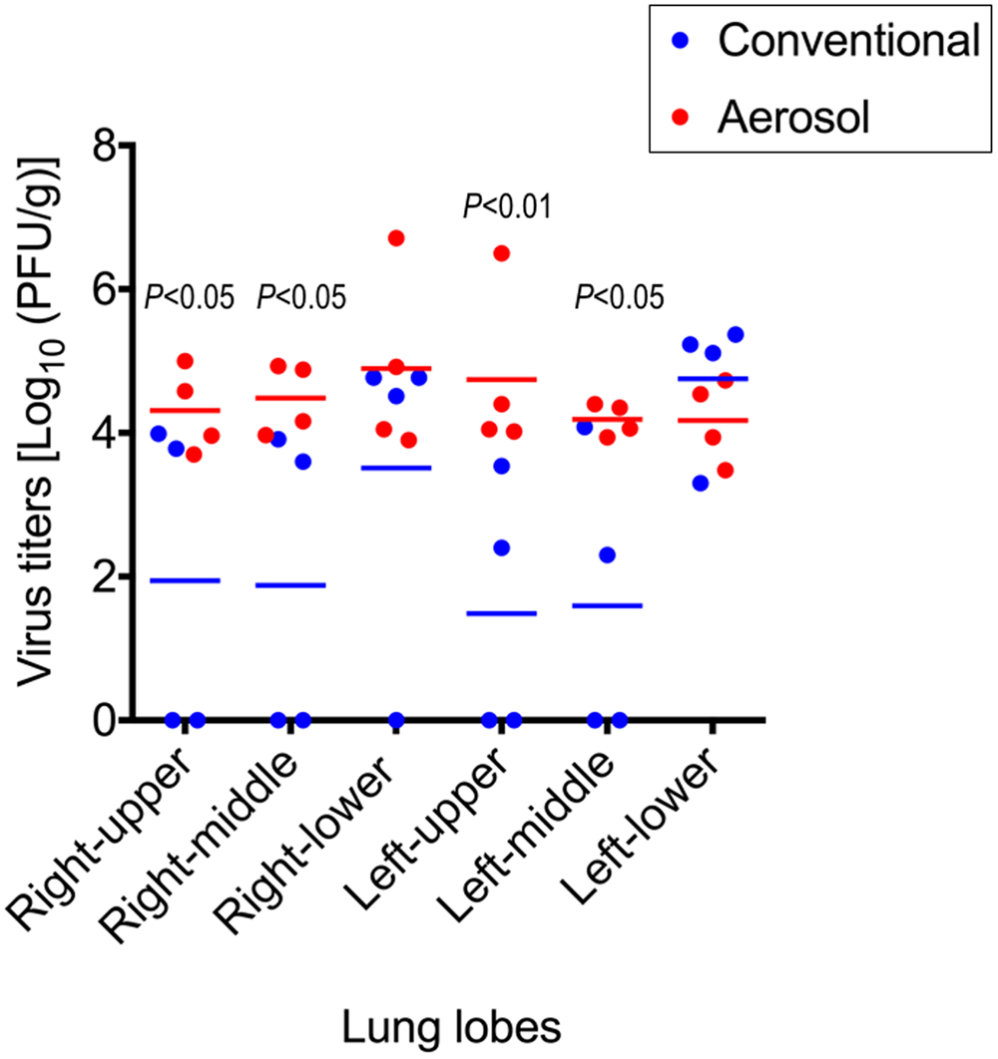
Replication of H5N1 virus (VN3040) in the lung lobes of infected monkeys. Cynomolgus macaques were inoculated with 4 ml of a 10^7^ PFU/ml solution of VN3040 through the aerosol route or conventional multi-site routes (i.e., intranasal, intraoral, intraocular, and intratracheal routes) by using the aerosol or conventional method, respectively. Four animals from each group were euthanized on day 4 post-infection for virus titration. Red and blue horizontal bars show the mean titers for the aerosol and conventional method groups, respectively. In the aerosol method group, the virus titers in some of the lung lobes were significantly higher than those in the conventional method group on day 4 post-infection (*p* < 0.05 in the right-upper, right-middle, and left-middle lobes, and *p* < 0.01 in the left-upper lobe). Detection limit = 1.3 log_10_ PFU/ml/tissue sample.

Interestingly, the virus distribution in the lower respiratory tract differed between the conventional and aerosol method groups. VN3040 virus replicated efficiently in all of the lung lobes of the infected animals in the aerosol method group, whereas no virus was recovered from some of the lung lobes of some animals in the conventional method group (Table 2). The mean virus titers in right upper, right middle, left upper, and left middle lobes of the lungs of the infected animals in the aerosol method group were significantly higher than those of the infected animals in the conventional method group [the mean virus titers in the aerosol method group were 4.31, 4.49, 4.74, and 4.19 log_10_(PFU/g), whereas those in the conventional method group were 1.94, 1.88, 1.49, and 1.60 log_10_(PFU/g), respectively] (Fig. 2). In contrast, VN3040 replicated well in the right- and left-lower lung lobes of the infected animals in the conventional method group [the virus mean titers were 3.51 and 4.75 log_10_(PFU/g), respectively]. Presumably, the introduction of virus as a liquid via the intratracheal and intranasal routes led to a large influx of virus solution into the right- and left-lower lobes. We assume that this also occurs in ferrets because we often observe similar macroscopic pathological changes in the lower lung lobes of infected ferrets (unpublished data).

Taken together, these findings demonstrate that a productive H5N1 virus infection in NHPs can be induced via the aerosol route by using the commercial ultrasonic nebulizer NE-U17, and inoculation of NHPs with aerosolized H5N1 virus can result in the development of virus infection across a large area of the lower respiratory tract and the distribution of virus in all of the lung lobes of the infected animals, whereas inoculation via the conventional method can result in some lung lobes not being exposed to or infected with virus.

### Histopathologic changes in the respiratory tract of animals infected with H5N1 virus via the conventional and aerosol methods

**Figure 3:**
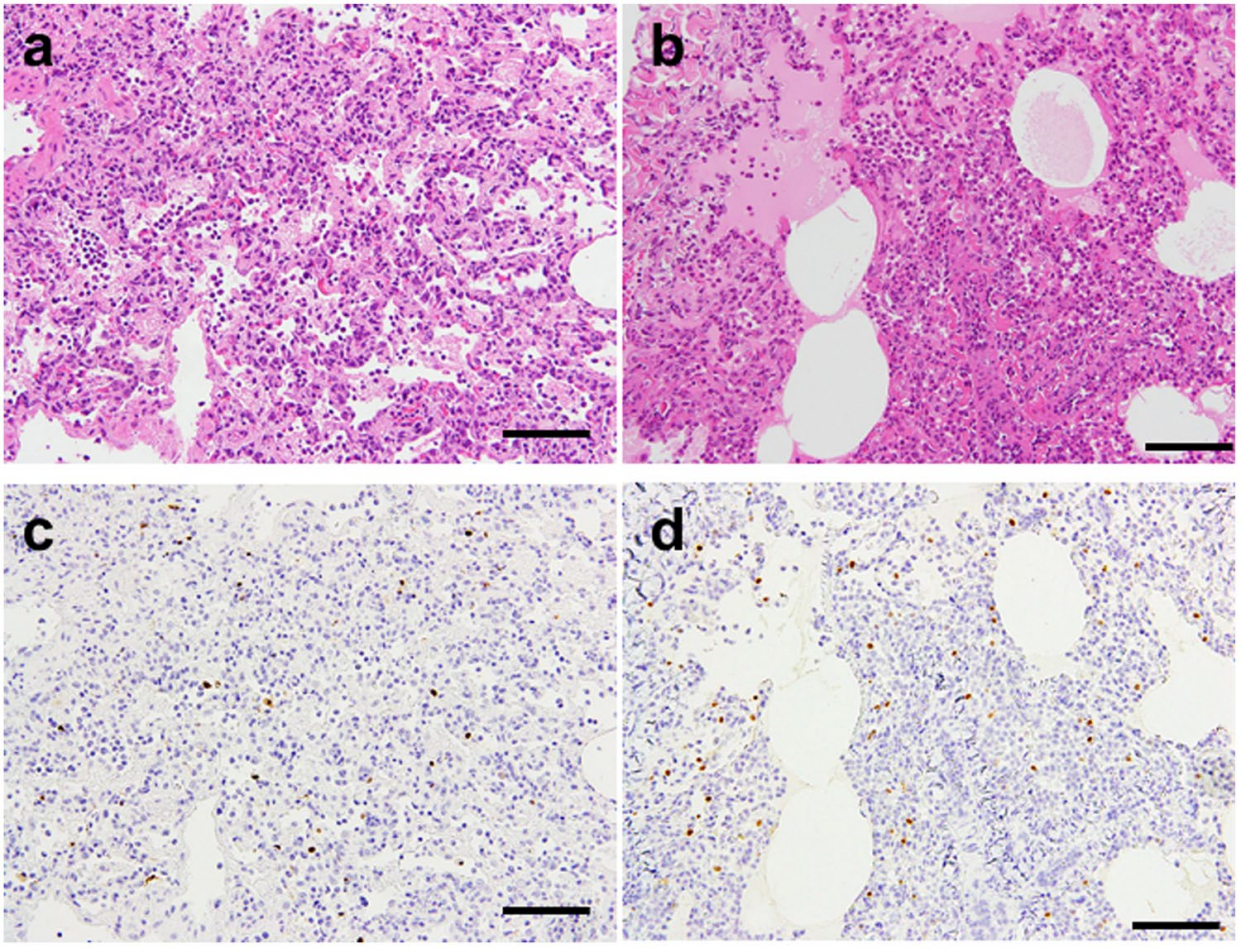
Pathological findings in the lungs of infected monkeys. Shown are representative pathological findings in the lungs of monkeys infected with VN3040 by using the conventional (**a**,**c**; right-lower lobe of animal #3–2, whose pathologic score and score of detection of antigen-positive cells were 5 and 3+, respectively) or aerosol (**b**,**d**; right-middle lobe of animal #3–3, whose pathologic score and score of detection of antigen-positive cells were 5 and 3+, respectively) method at day 4 post-infection with hematoxylin-eosin (HE) staining (**a**,**b**) and immunohistochemistry for influenza viral antigen (NP) (**c**,**d**). The animal ID numbers correspond to those shown in Tables 1, 2, and 3. Scale bar is 100 μm.

Next, we compared histopathological changes in the infected animals in the aerosol method group with those in the conventional method group. For this histopathologic analysis, we focused on two animals for each group in which virus was recovered from a variety of organs (i.e., #3–1 and #3–2 from the conventional method group, and #3–3 and #3–4 from the aerosol method group). Nasal cavity, trachea, lung, epiglottis, heart, liver, spleen, kidney, small intestine, large intestine, and brain (i.e., olfactory bulb, cerebrum, cerebellum, and brainstem) were examined using hematoxylin-and-eosin-stained sections obtained from the selected animals. We also examined the distribution of influenza virus antigen by immunohistochemistry. We found no obvious changes and no viral antigens in most of the tissues tested, except for the tracheal and lung tissues. In the trachea, we observed mild inflammatory cell infiltration but no virus-antigen-positive cells. Pulmonary edema and inflammatory cell infiltration into the alveolar spaces were observed in all of the infected animals tested. The degree of inflammation, which had spread from around the bronchi, was different for each part of the lungs of each animal. The total pathologic scores appear to be higher in the animals infected by the conventional method than those by the aerosol method, although no statistical analysis was conducted because of the small number of animals used (Table 3). The parts of the lung section with a pathologic score of 5 are shown in Fig. 3. Neutrophils and mononuclear cell infiltration with fibrinous exudates were observed in the interstitial areas and alveolar spaces. Edema and desquamation of alveolar epithelial cells into the alveolar spaces were also observed. These observations are identical to those reported in human patients who died as a result of H5N1 infection^20–23^. The conventional method group showed more severe inflammation in the lungs compared with the aerosol method group (Table 3). All animals showed virus-antigen-positive signals in the bronchial and alveolar epithelium (Table 3).

**Table 3:** Pathologic scores and detection of influenza virus antigen in animals infected with H5N1 virus via the conventional or aerosol method^a^.

Animal ID	Conventional method						Aerosol method					
	#3–1			#3–2			#3–3			#3–4		
	Pathologic score^b^	Detection of influenza virus antigen^c^		Pathologic score	Detection of influenza virus antigen		Pathologic score	Detection of influenza virus antigen		Pathologic score	Detection of influenza virus antigen	
		bronchial epithelium	alveolar epithelium		bronchial epithelium	alveolar epithelium		bronchial epithelium	alveolar epithelium		bronchial epithelium	alveolar epithelium
Lung (upper right)	5	1+	3+	5	1+	2+	5	1+	2+	4	1+	1+
Lung (middle right)	5	ND	2+	4	ND	3+	5	1+	3+	4	1+	2+
Lung (lower right)	5	ND	3+	5	ND	3+	4	ND	2+	3	1+	2+
Lung (upper left)	4	ND	2+	5	ND	2+	3	ND	2+	5	1+	2+
Lung (middle left)	5	ND	3+	4	1+	2+	3	1+	3+	3	1+	2+
Lung (lower left)	5	ND	3+	5	ND	3+	3	ND	2+	3	ND	2+

^a^Cynomolgus macaques were inoculated with 4 ml of a 10^7^ PFU/ml solution of VN3040 virus by using the conventional method (through a combination of intratracheal, intranasal, ocular, and oral routes) or via the aerosol method by using a nebulizer.

^b^Pathologic severity scores for infected animals. To represent comprehensive histological changes, respiratory tissue samples were evaluated by scoring pathologic changes. Pathologic scores were determined for each lung lobe (i.e., upper right, middle right, lower right, upper left, upper middle, and lower left lobes) of each animal in each group (6 lobes for each of 2 monkeys/group on day 4 post-infection) by using the following scoring system: 0 = no apparent changes; 1 = minimal changes or bronchitis–bronchitis; 2 = bronchitis–bronchiolitis (>50% of sections examined) and/or slight alveolitis; 3 = mild inflammation including infiltration of neutrophils, monocytes/macrophages, or lymphocytes; 4 = moderate inflammation (>50% of sections examined); 5 = marked inflammation including edema and fibrin transudation; 6 = severe inflammation (>50% of sections examined).

^c^Detection of influenza virus antigen in each tissue sample section: 1+, 1–10 virus-antigen-positive cells found; 2+, 11–101 virus-antigen-positive cells found; 3+, >101 virus-antigen-positive cells found.

^d^ND, not detected.

## Discussion

The use of animal models is essential for studying the biological properties of influenza viruses such as pathogenicity, replicative ability, and disease outcome. In most cases, intranasal administration with a liquid containing the virus is used to infect animals with influenza virus; however, a liquid inoculum does not reflect the natural influenza virus infection. Recently the aerosol inhalation method has been tested in several mammalian models, including mice, ferrets, and NHPs due to its resemblance to human exposure to influenza virus. Here, we inoculated NHPs with a highly pathogenic H5N1 influenza virus via the aerosol route and showed that aerosol infection did not affect clinical outcome, but did cause more widespread infection throughout the lower respiratory tract compared with the conventional approach. The uniform infection in all of the lung lobes and the bronchial epithelium afforded by the aerosol infection system offers an advantage in some experimental settings. For example, when researchers take lung tissues for multiple purposes (e.g., virus titration, histopathology, and various OMICs analyses), they need to take samples from different parts of the lungs. Therefore, infection via the aerosol route would minimize experimental variability.

Recently, Wonderlich *et al*.^15^ demonstrated that aerosol infection of NHPs with a highly pathogenic H5N1 avian influenza virus caused fulminant pneumonia that rapidly progress to acute respiratory distress syndrome, and some of the infected animals died or were humanely sacrificed due to respiratory failure. The same phenomenon has also been observed in the ferret model^9–11^; however, findings by Wonderlich *et al*. are not consistent with our findings here. The differences in disease outcomes between their study and ours may be caused by the different conditions used for the aerosol infection; that is, they used a head-only exposure chamber (into which the NHP’s head was inserted), which allowed the animals to inhale the aerosolized H5N1 virus with an exposure duration ranging from 15 to 34 min. In contrast, we used a standard inhalation mask, which is not a tight-fitting mask and allows the animals to breathe the aerosol mist through the nose and mouth spontaneously; the exposure duration was approximately 5 min, potentially leading to virus infection with a lower dose compared to Wonderlich’s study. In addition, other parameters, such as origin, gender, and age of the macaques, as well as the conditions for virus stock generation (i.e., egg-grown virus^15^ vs. MDCK-grown virus (our study), which could affect the resulting infectivity of the propagated virus), may have influenced the experimental results. Since it is difficult to standardize the parameters for NHP studies, we should pay attention to the experimental conditions when comparing results across different laboratories.

In summary, here we established a system for aerosol infection of NHPs with highly pathogenic H5N1 influenza virus. We found that experimental infection with aerosolized H5N1 virus led to a wider distribution of virus in the lower respiratory tract compared with liquid virus inoculation via the conventional method. However, no difference in clinical outcome was observed between the two inoculation methods.

## Methods

### Viruses

A highly pathogenic H5N1 avian influenza virus, A/Vietnam/UT3040/2004 (H5N1; VN3040), was isolated in our laboratory from the same human specimen from which A/Vietnam/1203/2004 (H5N1; VN1203) was isolated. There are no amino acid differences between VN3040 and VN1203 although there are two nucleotide differences, one in the NA gene at position 143 and the other in the M gene at position 250 (in both cases, VN3040 has adenine at these positions and VN1203 has guanine).

All experiments with VN3040 virus was performed in enhanced biosafety level 3 (BSL3) containment laboratories at the University of Tokyo and Kyoto University, Japan, which are approved for such use by the Ministry of Agriculture, Forestry, and Fisheries, Japan.

### Cells

Madin-Darby canine kidney (MDCK) cells were maintained in Eagle’s minimal essential medium (MEM) containing 5% newborn calf serum. The genetic origin of the MDCK cells was confirmed by DNA fingerprinting (using random amplified polymorphic DNA) conducted by Takara Bio Japan. MDCK cells were used for plaque assays to titrate viruses.

### Experimental infection of nonhuman primates

Two- to three-year-old male cynomolgus macaques, weighing 2.0–3.0 kg and serologically negative by neutralization assays for currently circulating human influenza viruses, were obtained from Cambodia (obtained from Japan Laboratory Animals, Inc., Tokyo, Japan). All experiments with macaques were performed in accordance with the Regulation on Animal Experimentation Guidelines at Kyoto University and were approved by the Committee for Experimental Use of Nonhuman Primates in the Institute for Virus Research, Kyoto University.

For the aerosol inoculation (aerosol method group), animals were anesthetized with ketamine via intramuscular injection and, by using the ultrasonic nebulizer NE-U17 (Omron Healthcare Co, Ltd, Japan), 4 ml of 10^7^ PFU/ml of virus was aerosolized and administered to each animal via an inhalation mask, which is not a tight-fitting mask and allows the individual to breathe the aerosol mist through the nose and mouth spontaneously. For virus inoculation through multiple routes, that is, the conventional method (the conventional method group), animals were anesthetized with ketamine via intramuscular injection and inoculated with a suspension containing a total of 4 × 10^7^ PFU of virus through a combination of the intratracheal (2.2 ml), intranasal (0.4 ml per nostril), ocular (0.1 ml per eye) and oral (0.4 ml each to the left and right tonsil) routes. Body temperature was monitored at 0, 1, 3, and 4 days post-infection by use of a rectal thermometer.

At the indicated timepoints post-infection, two or four macaques per group were euthanized for virologic and pathologic examinations. The virus titers in various organs and nasal swabs were determined by using plaque assays in MDCK cells.

### Pathologic examination

Animal tissues were fixed in 10% phosphate-buffered formalin for pathologic examination. They were then processed for paraffin embedding and cut into 3-µm-thick serial sections. One section from each tissue sample was subjected to standard hematoxylin-and-eosin staining, while another was processed for immunohistochemistry with a mouse monoclonal antibody for type A influenza virus NP antigen that reacts comparably with all of the test viruses. This antibody was produced in our laboratory and used for immunohistochemistry at 1:1000 dilution. Specific antigen-antibody reactions were visualized by use of 3,3′-diaminobenzidine tetrahydrochloride staining and a Dako EnVision system (Dako Co. Ltd., Tokyo, Japan).

### Statistical analysis

For statistical analyses, we used R (www.r-project.org) and lme4^24^ to fit a linear mixed effects model to the virus titer data. Different models were fitted for each biological question, namely, (i) the differences in virus titers between the groups over time (Table 1), and (ii) infection of different organs (Table 2), and infection of different lung parts (Table 2 and Fig. 2). In the first case, we used as fixed effects the different groups (i.e., the aerosol or conventional infection methods), and the time of the measurement. In the second case, we used as fixed effects the different groups (i.e., the aerosol or conventional infection methods), and the different organs in which the virus titers were measured. In both models, as random effects, we had intercepts for the different animals in the study. Finally, we used the lsmeans package^25^, to compare the groups for each model separately, and the *p*-values were adjusted using Holm’s method. *P*-values were considered significant if they were less than 0.05.

### Biosafety Statement

All recombinant DNA protocols were approved by the Subcommittee on Living Modified Organisms of the University of Tokyo and Kyoto University, and, when required, by the competent minister of Japan. All experiments were approved by the respective committees at the University of Tokyo and Kyoto University.

All experiments with H5N1 viruses were performed in enhanced biosafety level 3 laboratories at the University of Tokyo (Tokyo, Japan), and Kyoto University (Kyoto, Japan), which are approved for such use by the Ministry of Agriculture, Forestry and Fisheries, Japan. Staff working in enhanced BSL-3 and BSL-3Ag wear disposable overalls and powered air-purifying respirators. The enhanced BSL-3 facilities at the University of Tokyo and Kyoto University include controlled access, effluent decontamination, negative air-pressure, double-door autoclaves, HEPA-filtered supply and exhaust air, and airtight dampers on ductwork connected to the animal cage isolators and biosafety cabinets. The structure is pressure-decay tested regularly. All personnel complete biosafety and BSL-3 training before participating in BSL-3-level experiments.
